# PD-1/PD-L1 expression on CD^4+^ T cells and myeloid DCs correlates with the immune pathogenesis of atrial fibrillation

**DOI:** 10.1111/jcmm.12467

**Published:** 2015-03-26

**Authors:** Li Liu, Qiangsun Zheng, Jun Lee, Zhiqiang Ma, Qiming Zhu, Zhiquan Wang

**Affiliations:** a Department of Cardiology of Tangdu Hospital, Fourth Military Medical UniversityXi'an, China; b Department of Cardiology of PLA 161 HospitalWuhan, China

**Keywords:** Programmed death-1, programmed death-ligand 1, atrial fibrillation, myloid dendritic cell

## Abstract

Although immuno-inflammatory response contributes to pathogenesis of AF, molecular and cellular mechanism in this process remains poorly understood. Recently, increasing evidence suggests that Programmed death-1 (PD-1)/PD-1 ligand (PD-L) pathway may be a potential pathway participating in AF pathogenesis. In this study, we detected the PD-1 and PD-L1, 2 expression on peripheral blood function cells by flow cytometry in 91 atrial fibrillation (AF) patients and 35 healthy volunteers. The expression of PD-1 on CD^4+^ T cells and PD-L1 on myeloid dendritic cells (mDCs) in AF patients is significantly down-regulated compared with healthy volunteers. In addition, the extent of PD-1/PD-L1 down-regulation is closely related with AF burden. More importantly, Allogeneic mixed leukocyte reactions (MLR) shows that the mDCs PD-L1 down-regulation is associated with increased T cell (CD^4+^ and CD^8+^) proliferation, increased type 1 effector cytokines (IL-2 and IFN-γ) secretion, and decreased type 2 effector cytokine (IL-10) secretion. Then, PD-L1 up-regulation by the stimulation of IFN-α can significantly convert this representation. Collectively, our report suggest that T(CD^4+^)/mDCs-associated PD-1/PD-L1 pathway plays a key role in AF immune regulation. PD-1/PD-L1 down-regulation in AF patients promotes T cells function and may contribute to AF pathogenesis.

## Introduction

Atrial fibrillation (AF) is the most common arrhythmia, but the pathophysiological mechanism leading to this condition remains poorly understood. However, increasing evidence suggests the influence of an immune-mediated inflammatory response in AF pathogenesis 1. It has been established that serum or plasma inflammatory biomarkers including CRP and IL-6 2,3, and autoantibodies such as anti-M2 muscarinic receptor autoantibodies 4,5, are elevated in AF patients. Furthermore, studies indicate that activated T-lymphocytes infiltrate the endomyocardial in AF patients 6–8. Together, these observations suggest that the immune response contributes to AF pathogenesis. Nevertheless, there is insufficient knowledge to define the molecular and cellular mechanisms of the immune response in the AF process.

Programmed death-1 (PD-1, CD279) is a CD28 homolog and costimulatory molecule expressed on CD^4+^ and CD^8+^ T cells, NK cells, B cells and monocytes upon activation. It is engaged primarily by its ligands PD-L1 (B7-H1, CD274) and PD-L2 (B7-DC, CD273) 9,10. PD-L1 is widely expressed on immunocompetent cells such as T cells, B cells, dendritic cells (DCs) and macrophages in nonlymphoid organs, such as heart, lung, placenta, kidney and liver 11,12. However, PD-L2 expression is more restricted, and previous studies suggest that PD-L2 is inducibly expressed on DCs and macrophages 13,14. Recently, two studies found that PD-L2 is also expressed on CD^4+^ and CD^8+^ T cells 15,16. Increasing evidence suggests that the PD-1/PD-L pathway inhibits mainly T and B cell proliferation and cytokine production 17,18. Owing to its critical inhibitory function in immune regulation, the PD-1/PD-L pathway has been the focus of a number of recent studies. It is noteworthy that in the heart, PD-L1 and PD-L2 are expressed at high levels 10. Meanwhile, accumulating evidence implicates PD-1/PD-L pathway in the pathogenesis of certain cardiovascular diseases, including atherosclerosis 19,20, immune-mediated myocarditis 21,22 and dilated cardiomyopathy (DCM) 23,24. However, it remains unclear whether the PD-1/PD-L pathway influences immune regulation in AF.

In this study, we examined the expression profiles of PD-1 on T cells and PD-L1 and 2 on T cells, myeloid dendritic cells (mDCs) and macrophages in patients with paroxysmal and persistent AF, to assess their roles in AF pathogenesis. We found that PD-1/PD-L1 expression is substantially down-regulated on CD^4+^ T cells or mDCs in AF patients, and that the extent of the down-regulation is closely related with the AF burden. Additionally, PD-1/PD-L1 down-regulation significantly enhanced CD^4+^ and CD^8+^ T cell proliferation and influenced cytokines secretion *in vitro*. Our study indicates that the PD-L1 may be induced by IFN-α. We further up-regulated expression of PD-L1 on mDCs by IFN-α treatment and found that mDC-mediated T cell proliferation was suppressed and the pattern of cytokine secretion was converted. This suggests that PD-1/PD-L1 down-regulation on CD^4+^ T cells or mDCs may modulate immune regulation and play a role in AF pathogenesis.

## Materials and methods

### Patients

Between May 2010 and January 2012, a total of 91 patients with a diagnosis of AF were enrolled in the Department of Cardiology of Tangdu Hospital, Xi'an, China. The AF patients were divided into two groups: the paroxysmal AF group (*n* = 42) and the persistent AF group (*n* = 49). The diagnosis of AF was adopted in the ACC/AHA/ESC 2006 guidelines on AF 25. Thirty-five healthy volunteers with no history of arrhythmias who were undergoing a regular routine clinical examination for a health certificate served as control group. Exclusion criteria included: infectious diseases, immunological diseases, anti-inflammatory or immunosuppression treatment, recent trauma and surgery, coronary heart disease, myocardiopathy, rheumatic heart disease, valvular heart disease, heart failure, chronic lung diseases, hepatic and renal diseases, and cancer. The results of clinical histories, electrocardiogram and conventional biochemical tests were collected for study. The 42 paroxysmal AF patients were followed up 3 months and further divided into two groups. The occasional paroxysms group (group O) consisted of 17 patients who fulfilled the following criteria: (*i*) episodes of paroxysmal AF lasting less than 6 hrs each and (*ii*) a sum of paroxysmal AF lasting largely less than 30% of the total time. The frequent paroxysms group (group F) consisted of 15 patients with at least one of the following characteristics: (*i*) a total time of recurrent paroxysmal AF lasting largely longer than 30% of the total time and (*ii*) at least one episode of AF lasting more than 6 hrs. The other 10 patients who did not meet the above criteria were excluded. The time of paroxysms was mainly judged by ECG, holter monitor or symptoms of palpitations. This method of grouping can depict the arrhythmia burden and the risk for adverse events 26. People informed consent was obtained from each patient and control subject, and the study was approved by the Ethical Committee of the Fourth Military Medical University.

### Sample preparation and peripheral blood mononuclear cell isolation

Whole blood sample from each patient was collected by venepuncture of an antecubital vein at day 1 of hospitalization, with respect to concentrations of serum cholesterol, C-reactive protein (CRP), lymphocytes and white blood cell counting according to routine protocols. Peripheral blood mononuclear cells (PBMCs) were isolated from fresh heparinized blood by Ficoll–Hypaque (Sigma-Aldrich, St. Louis, MO, USA) density gradient centrifugation, and were viably frozen in 80% foetal calf serum (FCS; Gibco, Grand Island, NY, USA), 10% dimethylsulphoxide and 10% RPMI 1640 medium (Gibco) in liquid nitrogen for subsequent analysis.

### Flow cytometry

The following monoclonal antibodies (mAbs) were used: CD3-FITC (isotype, mouse IgG2a, κ), CD8-PE (isotype, mouse IgG2a, κ), CD4-APC (isotype, mouse IgG1, κ), PD-1-PE-cy7 (isotype, mouse IgG1, κ), PD-L1-PE (isotype, mouse IgG1, κ), PD-L1-PE-cy7 (isotype, mouse IgG1, κ), PD-L2-PE (isotype, mouse IgG1, κ), lineage-1-FITC, HLA-DR-PerCP (isotype, mouse IgG2a, κ), CD11c-APC (isotype, mouse IgG1, κ), CD14-APC (isotype, mouse IgG2a, κ). All Abs were purchased from BD Biosciences (San Jose, NJ, USA). PD-1 expression on T cells, PD-L1 and PD-L2 expression on mDCs, macrophages and T cells were measured, respectively, using fresh heparinized peripheral blood. In brief, blood samples were stained with a mixture of Abs (CD3-FITC, CD8-PE, CD4-APC, PD-1-PE-cy7, PD-L1-PE, PD-L1-PE-cy7, PD-L2-PE, lineage-1-FITC, HLA-DR-PerCP CD11c-APC, CD14-APC), as well as isotypecontrol antibodies, for 30 min. at 4°C. The cells were then processed with FACS lysing solution (BD Pharmingen, San Diego, CA, USA) to remove RBC, fixed and analysed by four-colour flow cytometry on a FACSCalibur (BD Biosciences). A total of 1 × 10^5^ cells were counted in each analysis. The results were processed with FCS express V3 software and described with the percentage of positive cells.

### Preparation of T cells/mDCs and PD-L1 expression

CD^4+^ T cells were positively isolated from PBMCs using the CD4 Positive Isolation Kit (Dynal Biotech). MDCs were obtained from PBMCs by CD19-negative selection, followed by CD1c-positive selection using the CD1c (BDCA-1) Dendritic Cell Isolation Kit (Miltenyi Biotech) with a MidiMACS separator unit. All procedures were performed according to the manufacturer's instructions. CD^4+^ T cells or mDCs purity exceeded 90%. Unless otherwise stated, freshly isolated cells were carefully collected and suspended in RPMI 1640 medium containing 10% FCS, 100 U/ml penicillin and 100 μg/ml streptomycin. MDCs were treated for 72 hrs in a 96-well plate in RPMI 1640 medium containing 10% FCS only or with saturating doses of IFN-α (10,000 U/ml). Cells were collected at 0, 24, 48 and 72 hrs of *in vitro* cell culture. The expression of PD-L1 was analysed immediately on a FACSCalibur (BD Biosciences).

### Allogeneic mixed leukocyte reactions

Purified mDCs cultured with poly(I:C) (50 μg/ml; Sigma-Aldrich) were performed in 96-well flat-bottom culture plates (at 1 × 10^5^ cells/200 μl) for 24 hrs. In some cases, IFN-α (10,000 U/ml) was added in the culture medium. On the following day, mDCs were mixed at different concentrations (1/5, 1/10, 1/20, 1/40 and 1/80) with purified CD4 or CD8 T cells (1 × 10^5^ cells/200 μl) from a healthy individual in the presence of 2 μg/ml anti-B7-H1 (eBiosciences, San Diego, CA, USA) or control antibodies (mouse IgG1, κ; eBiosciences) for 5 days. Cells were pulsed with 1 μCi/well of [^3^H]thymidine (Amersham Biosciences, Uppsala, Sweden) for 18 hrs before harvest. Before measurement of incorporated [^3^H]thymidine, part of the culture supernatants was collected for cytokine detection.

### ELISA for cytokines

Cytokine assessment was carried out using ELISA Kit (eBioscience) for three cytokines (IL-2, IL-10 and IFN-γ) of culture supernatants. All procedures were performed according to the manufacturer's instructions. Absorbance was measured on an automatic plate reader. The sensitivity of these ELISA kits were 1 pg/ml for IL-10, 2 pg/ml for IL-2 and 4 pg/ml for IFN-γ.

### Statistical analysis

All experiments were repeated three times. Quantitative data were expressed as mean ± SD, and categorical variables as absolute and relative frequencies (percentages). The statistic software SPSS 19.0 was employed for statistical processing. Continuous data were tested for normal distribution by the Kolmogorov–Smirnov test and were found to be suitable for parametric analyses. One-way anova was employed for comparing means of multiple groups, followed least significant difference (LSD) for paired comparison. The Mann–Whitney *U*-non-parametric test was used for differences between non-parametric analyses. To evaluate differences in categorical variables between study groups Pearson's chi-squares test was used. All *P*-values were two-tailed and values <0.05 were considered statistically significant.

## Results

### Characteristics of the study individuals

The characteristics of the study participants in this study are presented in Table1. There were no statistically significant differences among the three groups in the following parameters: age, gender, hypertension, hyperlipidaemia, diabetes, angiotensin-converting enzyme inhibitors use, angiotensin receptor blocking agents use, calcium channel blockers use, statins use, lymphocytes count and white blood cells count (all *P* > 0.05). β-blocker as heart rate-slowing agent was used significantly more in AF patients groups than control group (*P* < 0.001). AF patients groups had higher CRP levels compared with control group and CRP level in persistent AF group was further higher than paroxysmal AF group (all *P* < 0.001).

**Table 1:** Characteristics of the studied population

	Control (*n* = 35)	Paroxysmal AF (*n* = 42)	Persistent AF (*n* = 49)	*P*
Age (years)	64.1 ± 8.0	64.4 ± 9.7	67.3 ± 9.6	0.202
Men	22 (63%)	25 (60%)	28 (59%)	0.277
Hypertension	9 (26%)	18 (43%)	23 (47%)	0.128
Hyperlipidemia	7 (20%)	7 (17%)	6 (12%)	0.949
Diabetes	2 (6%)	3 (7%)	5 (10%)	0.618
Drugs				
ACE-I/ARB	8 (23%)	10 (24%)	20 (41%)	0.115
Statins	6 (17%)	5 (12%)	4 (8%)	0.456
β-blockers	5 (14%)	23 (55%)	29 (59%)	<0.001
CCBs	4 (11%)	10 (24%)	6 (12%)	0.225
WBC count (per μL)	6349 ± 1891	6768 ± 1859	6375 ± 1663	0.493
Lymphocytes (%)	31.4 ± 3.7	32.6 ± 4.3	31.4 ± 4.1	0.296
CRP (mg/dl)	0.18 ± 0.07	0.46 ± 0.20	0.56 ± 0.18*	<0.001

ACEI, angiotensin-converting enzyme inhibitors; ARB, angiotensin receptor blocking agents; CCBs, calcium channel blockers; WBC, white blood cells; CRP, C-reactive protein; *P*, probability of significance (difference among three groups)

* *P* < 0.001 persistent AF *versus* paroxysmal AF. *P*-values were calculated by Pearson's chi-squared test, anova and LSD.

### Cell surface expression of PD-1 on CD^4+^ and CD^8+^ T cells

**Figure 1:**
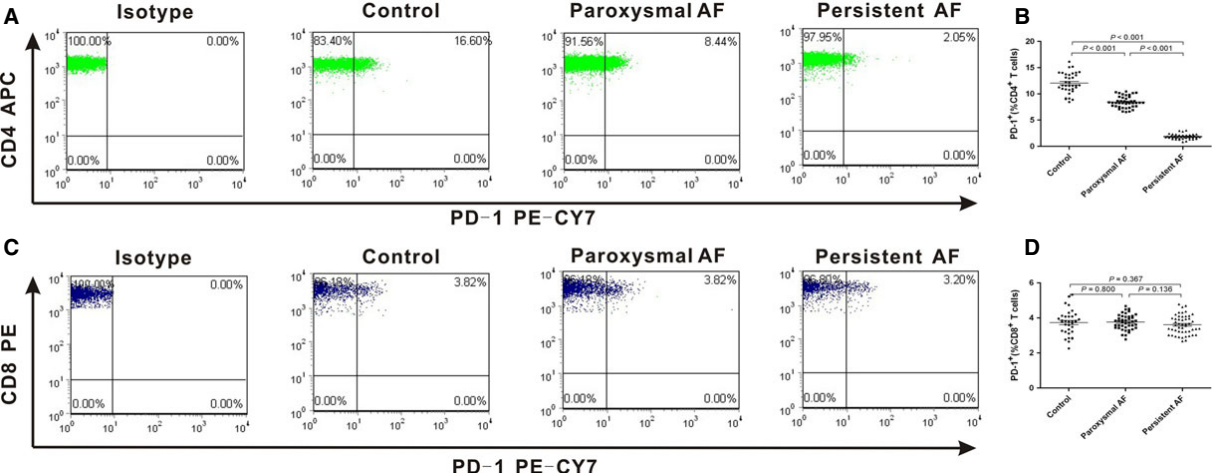
Expression of PD-1 on T cells from AF patients. Fresh heparinized peripheral blood were stained with a mixture of mAbs and analysed by flow cytometry. (A and C) Representative dot plots of PD-1 staining for studied individuals. (B and D) Percentages of PD-1+ CD^4+^ T cells and PD-1+ CD^8+^ T cells are shown respectively. •, control group; ▪, paroxysmal AF patient; ▴, persistent AF patient. Horizontal bars, mean values. Values of *P* are shown. *P*-values were calculated by anova and LSD.

To explore the surface expression characteristics of PD-1/PD-L on peripheral blood cells, we firstly detected PD-1 expression on CD^4+^ and CD^8+^ T cells in paroxysmal AF patients, persistent AF patients and control group. Representative dot plots of PD-1+ distinct subpopulations were shown in Figure1A and C. The results showed that PD-1+ distinct subpopulations of CD^4+^ T cells in AF patients was significantly decreased (*P* < 0.001) than healthy controls (12.04 ± 1.95). Compared the two groups, we found persistent AF patients (1.79 ± 0.32) expressed a lower level of PD-1 than paroxysmal AF patients (Fig.1B; 8.45 ± 1.07). However, there were no statistically differences in PD-1+ distinct subpopulations of CD^8+^ T cells among paroxysmal AF group (3.76 ± 0.43), persistent AF group (3.61 ± 0.54) and control group (Fig.1D; 3.73 ± 0.70).

### Cell surface expression of PD-L1, and PD-L2 on mDCs, macrophages and T cells

**Figure 2:**
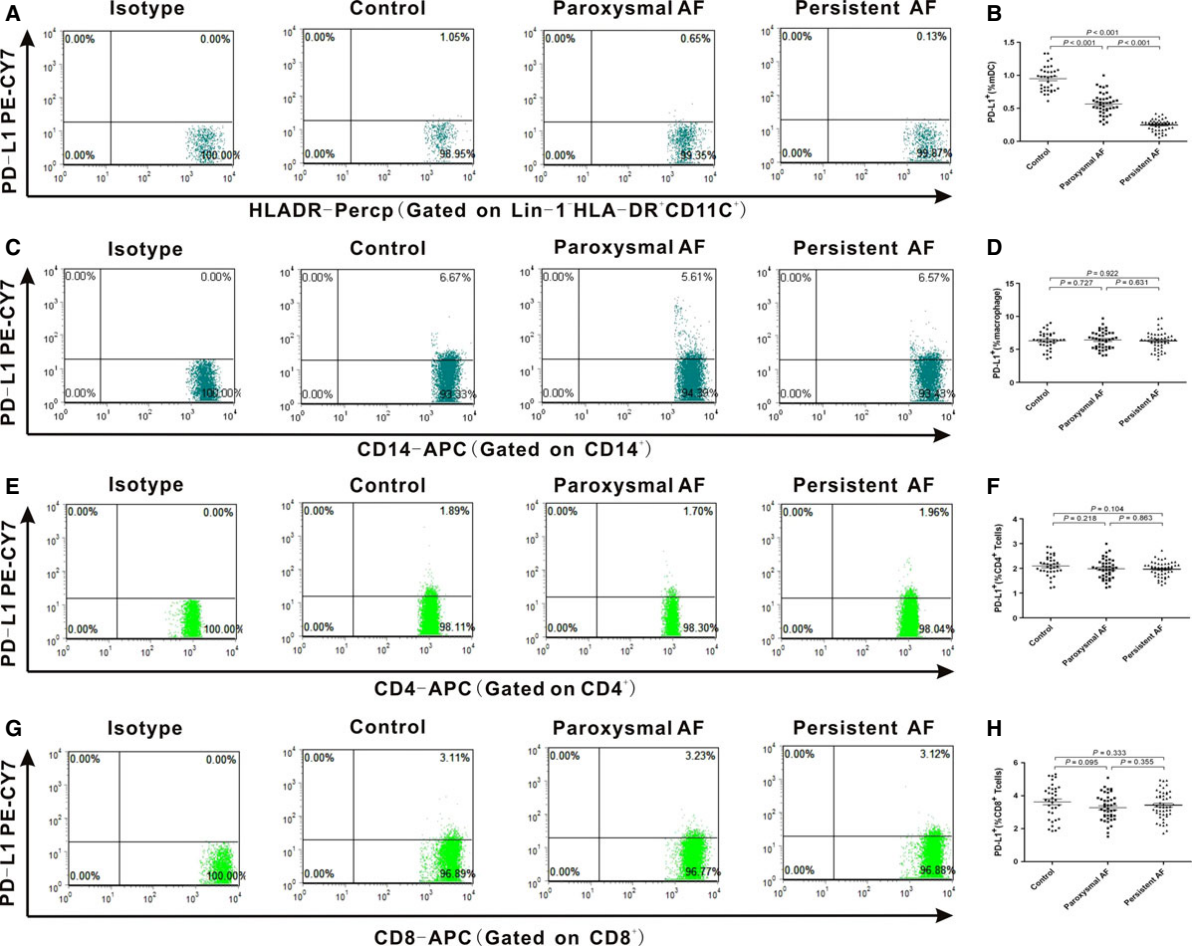
Expression of PD-L1 on mDCs, macrophages, CD^4+^ and CD^8+^ T cells from AF patients. Fresh heparinized peripheral blood were stained with a mixture of mAbs and analysed by flow cytometry. (A, C, E and G) Representative dot plots of PD-L1 staining for studied individuals. (B, D, F and H) Percentages of PD-L1+ mDCs+, PD-L1+ macrophages+, PD-L1+ CD^4+^ T cells and PD-L1+ CD^8+^ T cells are shown respectively. •, control group; ▪, paroxysmal AF patient; ▴, persistent AF patient. Horizontal bars, mean values. Values of *P* are shown. *P*-values were calculated by anova and LSD.

**Figure 3:**
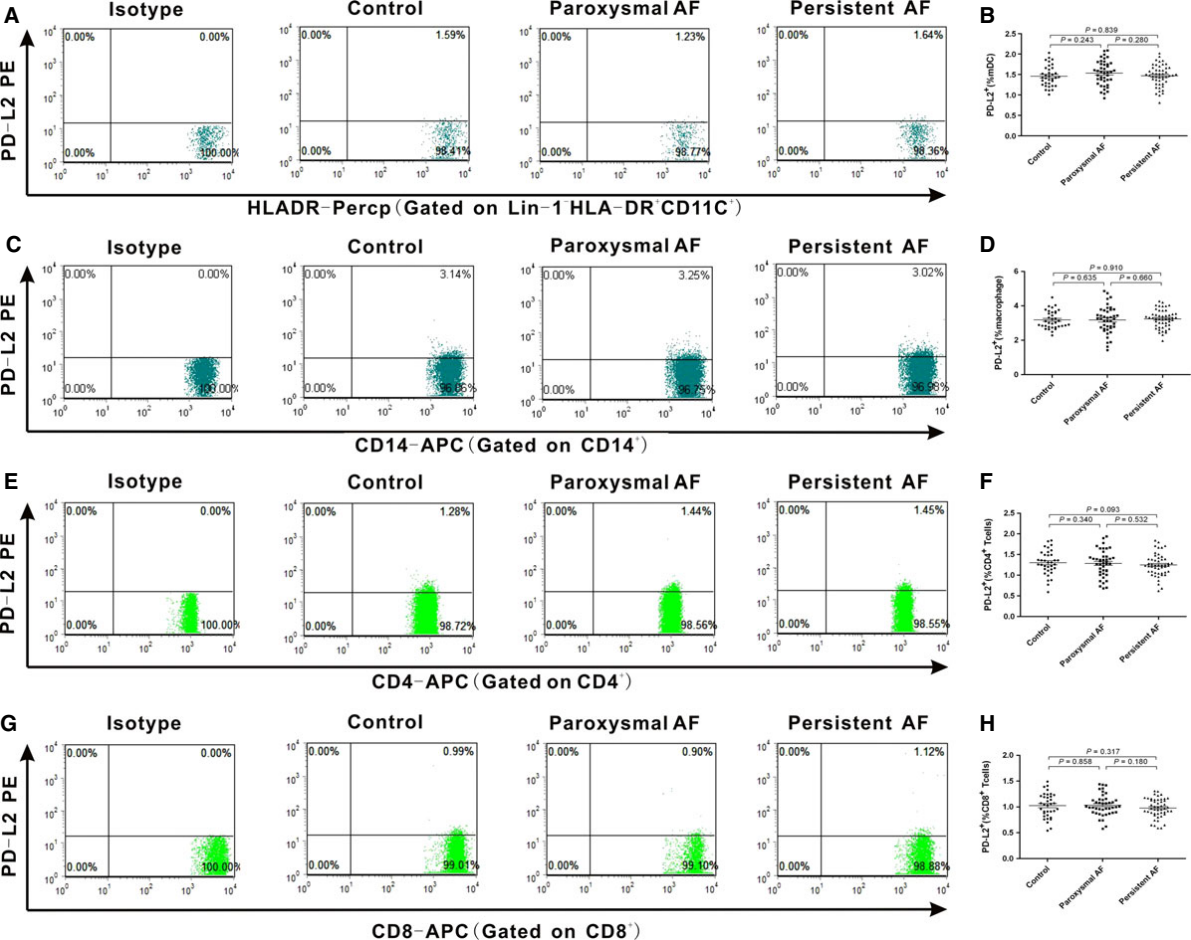
Expression of PD-L2 on mDCs, macrophages, CD^4+^ and CD^8+^ T cells from AF patients. Fresh heparinized peripheral blood were stained with a mixture of mAbs and analysed by flow cytometry. (A, C, E and G) Representative dot plots of PD-L2 staining for studied individuals. (B, D, F and H) Percentages of PD-L2+ mDCs+, PD-L2+ macrophages+, PD-L2+ CD^4+^ T cells and PD-L2+ CD^8+^ T cells are shown respectively. •, control group; ▪, paroxysmal AF patient; ▴, persistent AF patient. Horizontal bars, mean values. Values of *P* are shown. *P*-values were calculated by anova and LSD.

We further detected PD-L1 and PD-L2 expression on the function cells. Representative dot plots of PD-L1+ and PD-L2+ distinct subpopulations were shown in Figures2A, C, E, G and 3A, C, E, G. The results showed that PD-L1 expression on mDCs in AF patients was significantly down-regulated (*P* < 0.001) than healthy controls (0.95 ± 0.19), and persistent AF group (0.25 ± 0.08) was lower than paroxysmal AF group (Fig.2B; 0.57 ± 0.16). We found that there were no statistically differences in PD-L1 expression on macrophages, CD^4+^ and CD^8+^ T cells among the three groups (Fig.2D, F and H). As for the second PD-ligand, PD-L2 expression on mDCs, macrophages, CD^4+^ and CD^8+^ T cells in the three groups all had no statistically differences (Fig.3B, D, F and H). These down-regulated expression of PD-1 on CD^4+^ T cells and PD-L1 on mDCs in AF patients suggested that the PD-1/PD-L1 pathway involving in T(CD^4+^)/mDCs interactions might participate in the pathogenesis of AF.

### Results of follow-up study

**Figure 4:**
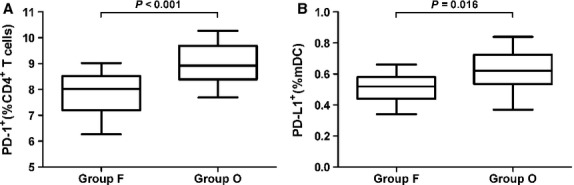
Expression of PD-1 on CD^4+^ T cells and PD-L1 on mDCs from group F and group O. The expression of PD-1 on CD^4+^ T cells and PD-L1 on mDCs were shown as a box with the 25th to 75th percentiles containing the median line and the lines showing the 1st to 99th percentiles. Values of *P* are shown. *P*-values were calculated by Mann–Whitney *U*-test.

We found that the frequency of AF episodes correlated with lower surface expression of PD-1/PD-L1 on peripheral blood mDC and CD^4+^ T cells. To further explore this phenomenon, we examined a paroxysmal AF group with a 3-month follow-up. We divided them into two subgroups (group F and group O) and measured PD-1/PD-L1 expression on CD^4+^ T cells and mDCs. The results showed that PD-1+ distinct subpopulations of CD^4+^ T cells and PD-L1+ distinct subpopulations of mDCs in group F (7.86 ± 0.77; 0.51 ± 0.09) were significantly lower than group O (9.00 ± 0.81; 0.61 ± 0.13; Fig.4A and B). Results of the follow-up study suggested that the extent of PD-1/PD-L1 down-regulation was closely related with the AF burden. In short, the frequency of AF episodes correlated with lower expression of PD-1/PD-L1 on CD^4+^ T cells and mDCs.

### IFN-α induce *in vitro* PD-L1 expression

**Figure 5:**
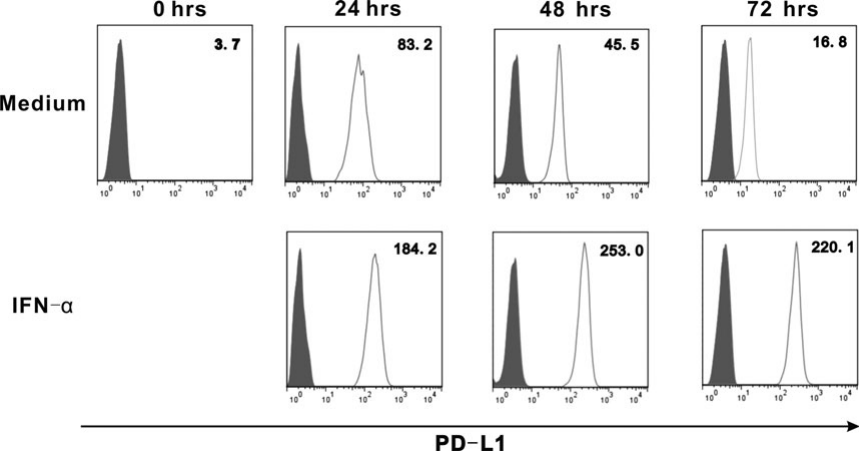
Induction of PD-L1 expression on mDCs by IFN-α *in vitro*. The isolated mDCs were cultured with medium only or 10,000 U/ml IFN-α in 96-well plates for 72 hrs. PD-L1 was stained with a control Ab (filled histograms) or PD-L1 mAb (open histograms). Numbers in histograms represent mean fluorescence intensity of PD-L1 expression. Data shown are one of three similar experiments.

It has been reported that IFN-α stimulates PD-L1 on mDCs, although the mechanism by which this occurs is unclear 27. To investigate IFN-α regulation of PD-L1 on mDCs, isolated mDCs were cultured with or without IFN-α stimulation. mDCs cultured in medium alone showed a slight increase in PD-L1 expression, peaking at 24 hrs before returning to background levels at 72 hrs (Fig.5). In contrast, IFN-α (10,000 U/ml) induced PD-L1 expression on mDCs in the first 24 hrs, with expression sustained up to 72 hrs.

### Influence of PD-L1 expression on mDCs on T cells proliferation

**Figure 6:**
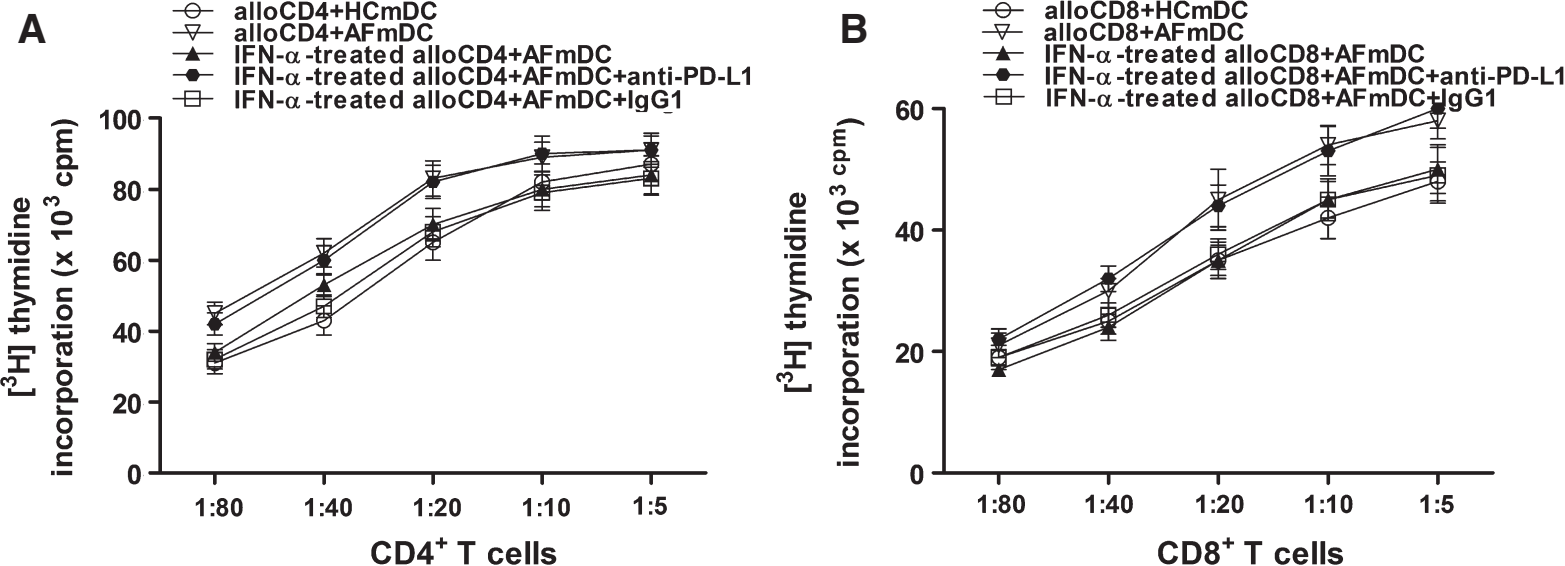
Effects of PD-1/PD-L1 pathway on T cell proliferation costimulated by mDCs. mDCs isolated from patients (*n* = 10) or healthy controls (*n* = 10) were cocultured at different ratios with CD^4+^ or CD^8+^ T cells from a normal individual (as the third party), with or without treatment of IFN-α. After 5 days at 37°C, proliferation of alloreactive T cells was assessed by pulsing the cultures with [^3^H] thymidine for the last 18 hrs. Results are expressed as mean cpm ± SD of triplicate wells. ○, healthy control; ▽, persistent AF patient; ▴, IFN-α-treated mDCs from patient groups; •, addition of anti-PD-L1 mAbs; and □, addition of isotype control IgG1.

In the allogeneic mixed leukocyte reactions (MLR), we cocultured mature mDCs from persistent AF patients and healthy volunteers at different ratios with T cells from a third healthy individual and studied how the decreased PD-L1 expression on mDCs in AF patients influence the T cells proliferation. The results showed that mDCs of AF patients (*n* = 10) were more efficient in inducing CD^4+^ and CD^8+^ T cell proliferation at all ratios tested compared with mDCs from controls (*n* = 10; Fig.6; all *P* < 0.05). It suggested that the T cell-stimulating capacity of mDCs from AF patients was enhanced. Then, we examined the effects of mDC-associated PD-L1 up-regulation on allogeneic T cell proliferation in the presence of IFN-α. We found that the upgrade capacity of mDCs from AF patients to stimulate CD^4+^ and CD^8+^ T cells proliferations was restored, leading to results similar to that of normal mDCs (Fig.6; all *P* < 0.05). Furthermore, we examined the effects on allogeneic MLR in the presence of anti-PD-L1. As expected, we found that the restored capacity of mDCs from AF patients to stimulate CD^4+^ and CD^8+^ T cells proliferations was up-regulated again by treated with anti-PD-L1 (Fig.6; all *P* < 0.05). These data suggested that the down-regulation of PD-L1 on mDCs appears to transmit stimulating signal to CD^4+^ and CD^8+^ T cells in AF patients, resulting in an increase of proliferation and PD-L1 up-regulation on mDCs can reduce the allostimulatory capacity of mDCs from AF patients.

### MDC-associated PD-L1 expression is involved in cytokine production by T cells and mDCs themselves

**Figure 7:**
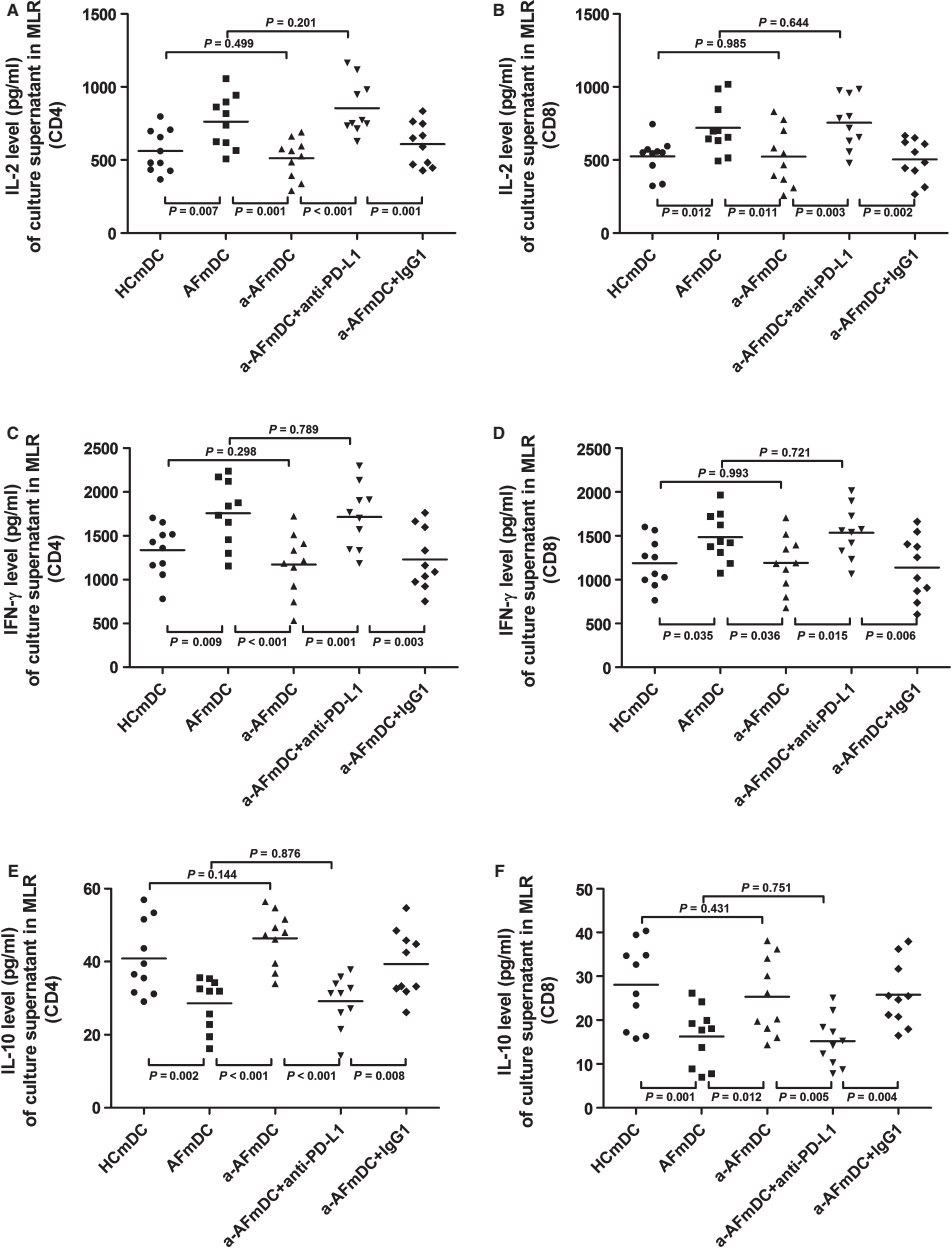
Up-regulation of PD-L1 can restore cytokine production by responsive cells in MLR. Data are expressed in pg/ml. •, healthy control; ▪, AF patients; ▴, IFN-α-treated mDCs from AF group; ▾, addition of anti-PD-L1 mAbs; and ♦, addition of isotype control IgG1. Horizontal bars, median values. Values of *P* are shown. *P*-values were calculated by Mann–Whitney *U*-test.

Cytokines are the key regulatory factors that modulate the strength of the T cell response and activation 28. We therefore examined the production of type 1 effector cytokines IL-2 and IFN-γ, and the type 2 effector cytokines IL-10, in culture supernatants of allogeneic MLR to examine whether PD-L1 expression influences cytokine production. We found that the mDCs with low-level PD-L1 from AF patients were more efficient at inducing IL-2 and IFN-γ secretion by both CD4 and CD8 T cells, than were healthy controls. We then stimulated PD-L1 expression on mDCs by IFN-α treatment and found that IL-2 and IFN-γ were both significantly decreased by T cells, which was similar to that observed for mDCs from healthy volunteers. Furthermore, we found that changes in cytokine production resulting from IFN-α treatment could be reversed by anti-PD-L1 mAbs treatment. We also examined production of IL-10, an anti-inflammation cytokine antagonistic to IL-2 and IFN-γ activity 29, and found that the IL-10 secretion pattern was opposite that observed with IL-2 and IFN-γ (Fig.7E and F). These observations suggest that the decrease of inhibitory signals by PD-L1 down-regulation on mDCs result in an increase of pro-inflammation cytokines (IL-2 and IFN-γ) secretion, and a decrease of anti-inflammation cytokines (IL-10) secretion. This is consistent with the idea that T cells proliferation seems to promote AF. These inhibitory signals can be mostly blocked by treating with IFN-α, further supporting the notion that PD-L1 down-regulation may be responsible for enhanced T cell responses and changed cytokines secretion.

## Discussion

AF is the most common sustained arrhythmia and has serious health consequences owing to haemodynamic impairment and thromboembolic events 30. However, the pathophysiological nature of AF is complicated and remains largely unknown. Recently, the link between inflammation and AF has been gaining attention 1 and many studies suggest that the immune response may contribute to this inflammatory process 2–8,31,32. Exploring the mechanism of the immune response in AF will be critical to further elucidating the pathophysiological mechanism of AF. This will be important for developing a therapeutic regimen to improve or terminate AF.

It has been well-demonstrated that in addition to the signal from the binding between MHC/peptide complex and T cell receptor which is called the first signal, the second signal that regulates the balance between stimulatory and inhibitory signals is even more important for effective immune response 10. The PD-1/PD-L signalling pathway (belonging to the B7:CD28 family) has been highlighted recently as important regulators for maintaining this critical balance. Several studies have also shown that the PD-1/PD-L pathway plays primarily a negative regulatory role in regulating T cell activation, proliferation and cytokine production 13,33. Additional studies further indicate that PD-1 ligation blocks TCR-mediated signalling by preventing phosphorylation and activation of protein kinase C 10,34. Meanwhile, the PD-1/PD-L pathway has been shown to play a pathogenic role in many immunoinflammatory diseases such as chronic intestinal inflammation 35, chronic inflammatory mucocutaneous disease 36, rheumatoid arthritis 37 and helicobacter pylori infection 38. However, it is unclear whether there are important inhibitory signals that also participate in AF, which is a putative immunoinflammatory disease. It is important to note that PD-L1 and PD-L2 expression is at high levels in the heart 10. This may imply that they play an essential role in the cardiovascular system, and in fact the PD-1/PD-L pathway has been shown to play at least a partial role in cardiovascular diseases. Using a mouse model, Gotsman *et al*. 19 has shown that the PD-1/PD-L pathway exerts a significant regulatory influence on the immune response and is associated with development of arterial disease. Grabie *et al*. 22 and Lucas *et al*. 21 have also revealed a critical role for the PD-1/PD-L pathway in controlling autoimmune cardiac injury. Given this, it will be interesting to explore the potential relationship between the PD-1/PD-L pathway and AF pathogenesis.

In this study, we comprehensively investigated the expression of PD-1 and PD-L1 and 2 on key regulatory cells including mDCs, macrophages, and CD^4+^ and CD^8+^ T cells in AF patients. Allogeneic MLR was carried out to explore the role of the T(CD^4+^)/mDCs-associated PD-1/PD-L1 pathway in immune regulation of AF. Our results suggest that down-regulation of PD-1/PD-L1 on CD^4+^ T cells or mDCs might, at least in part, take part in AF pathogenesis. Firstly, PD-1 expression on CD^4+^ T cells and PD-L1 on mDCs is down-regulated in AF patients compared with controls. Secondly, down-regulation of PD-1/PD-L1 was more significant in persistent AF patients, those considered to have a more pronounced inflammatory state compared with paroxysmal AF patients. This suggests that the extent of PD-1/PD-L1 down-regulation may be closely related with AF burden, which is further supported by the subsequent follow-up investigation. Thirdly, allogeneic MLR experiments indicate that low-level mDCs PD-L1 expression promotes T cell proliferation, suggesting that the PD-L1 levels may be linked with the inflammatory state. Cytokine secretion changed with mDC PD-L1 levels, further supporting this assertion. Collectively, PD-1/PD-L1 down-regulation on CD^4+^ T cells or mDCs may participate in the process of immune regulation and play a role in AF pathogenesis.

Regarding the underlying mechanisms about how PD-1/PD-L1 down-regulation modulates AF pathogenesis, we speculate on the following possibilities. Firstly, PD-1/PD-L1-associated T cell proliferation may contribute to atrial electrical remodelling. T cell proliferation may also increase secretion of cytokines, such as IL-6 and MCP-1, which may affect the contractility and electrical stability of myocytes differently 39. Furthermore, studies indicate that cytokine and inflammatory responses participate in atrial structural remodelling 39–41. Additional studies found that MRL mice with a PD-1 deficiency spontaneously developed severe myocarditis, which lead to deposits in the extracellular matrix and displayed massively enlarged hearts 21,42. Another study further demonstrated that because of cardiac troponin I autoantibodies, PD-1 deficient mice developed autoimmune DCM 23. Although these inferences are consistent with a role for the PD-1/PD-L1 pathway in AF pathogenesis, the underlying mechanisms need to be investigated further.

In this study, we investigated PD-L1 expression on several cell types, and mDCs have been proposed to be responsible for specific T cell regulation in AF. As a subpopulation of DCs, mDCs express elevated levels of MHC and a range of accessory molecules involved in cell migration, adhesion and co-signalling 43. Our study found that expression of inhibitory PD-L1 molecules on mDCs was significantly down-regulated in AF patients, whereas IFN-α expression induced PD-L1 on mDCs. We therefore used IFN-α to stimulate PD-L1 expression on mDCs isolated from AF patients in allogeneic MLR. Allogeneic MLR showed that AF mDCs with decreased PD-L1 were more effective in stimulating T cell proliferation and changing cytokine secretion, suggesting that mDCs-associated PD-L1 down-regulation tips the balance between inhibitory and stimulatory signals delivered to T cells in AF patients. Furthermore, we found that PD-L1 expression levels on AF macrophages were similar to that of controls. This might suggest that macrophages, an important immune cell, do not contribute to AF progression. Similarly, PD-L1 expression on CD^4+^ and CD^8+^ T cells in AF patients was not statistically different compared with controls, suggesting that PD-1/PD-L1 inhibitory signals between T-T cells may not participate in regulating T cells. Importantly, the expression of PD-L2 on mDCs, macrophages, and CD^4+^ and CD^8+^ T cells in AF patients was not statistically different compared with controls. Our study suggests that PD-L2 indeed is expressed on CD^4+^ T and CD^8+^ T cells, consistent with earlier studies 15,16. Although PD-L2 has been recognized to play a pivotal role in diseases such as asthma 44 and EAE 45, it is seemingly not involved in AF pathogenesis. Collectively, these results suggest a T(CD^4+^)/mDCs-associated PD-1/PD-L1-independent mechanism in AF regulation.

It is important to note that several limitations that need to be addressed regarding this study. First, to supporting the suggestion that the PD-L1 levels may be linked with the inflammatory state, more cytokines except IL-2, IL-10 and IFN-γ need to be examined. In addition, TNF-α as another important inflammatory cytokines may be not relevant to PD-L1 change in MLR (data not shown). The specific mechanisms is unclear and need further research. Second, although the PD-1/PD-L1 pathway appears to be important, additional co-inhibitory pathways such as those involving CTLA-4 and B7 interactions cannot be excluded in AF pathogenesis. Further study will be needed to determine whether the PD-1/PD-L1 pathway is the only mechanism or if it is just one that regulates the immune response in AF. Third, the specific role of the PD-1/PD-L1 pathway in AF is not clear and it remains unclear whether PD-1/PD-L1 down-regulation is a cause of AF, or merely a consequence.

In summary, the present study begins to shed light on the role of immune pathogenesis in AF, showing that PD-1 is down-regulated on CD^4+^ T cells and PD-L1 on mDCs in AF patients. The extent of PD-1/PD-L1 down-regulation was closely related with AF burden. Furthermore, PD-1/PD-L1 down-regulation could improve, at least in part, T cell proliferation and cytokine secretion change, which reflect the inflammatory state and contribute to AF pathogenesis. Therefore, PD-1/PD-L1 down-regulation on CD^4+^ T cells or mDCs participates in the pathogenesis of AF and up-regulating the expression of PD-1/PD-L1 molecules may represent a new therapeutic option for AF.
